# Self-Aligned Hierarchical ZnO Nanorod/NiO Nanosheet Arrays for High Photon Extraction Efficiency of GaN-Based Photonic Emitter

**DOI:** 10.3390/mi11040346

**Published:** 2020-03-26

**Authors:** Won-Seok Lee, Soon-Hwan Kwon, Hee-Jung Choi, Kwang-Gyun Im, Hannah Lee, Semi Oh, Kyoung-Kook Kim

**Affiliations:** 1 Department of Advanced Convergence Technology, Research Institute of Advanced Convergence Technology, Korea Polytechnic University, Gyeonggi-do 15073, Korea; dldnjstjr37@kpu.ac.kr (W.-S.L.); canwkd21@kpu.ac.kr (S.-H.K.); gmlwjd0889@kpu.ac.kr (H.-J.C.);; 2 Department of Nano & Semiconductor Engineering, Korea Polytechnic University, Gyeonggi-do 15073, Korea; rhkdrbs87@kpu.ac.kr; 3 Department of Electrical Engineering and Computer Science, University of Michigan, Ann Arbor, MI 48109, USA

**Keywords:** self-align, hierarchical nanostructures, ZnO nanorod/NiO nanosheet, photon extraction efficiency, photonic emitter

## Abstract

Advancements in nanotechnology have facilitated the increased use of ZnO nanostructures. In particular, hierarchical and core–shell nanostructures, providing a graded refractive index change, have recently been applied to enhance the photon extraction efficiency of photonic emitters. In this study, we demonstrate self-aligned hierarchical ZnO nanorod (ZNR)/NiO nanosheet arrays on a conventional photonic emitter (C-emitter) with a wavelength of 430 nm. These hierarchical nanostructures were synthesized through a two-step hydrothermal process at low temperature, and their optical output power was approximately 17% higher than that of ZNR arrays on a C-emitter and two times higher than that of a C-emitter. These results are due to the graded index change in refractive index from the GaN layer inside the device toward the outside as well as decreases in the total internal reflection and Fresnel reflection of the photonic emitter.

## 1. Introduction

The advancements in nanotechnology have facilitated the increased use of ZnO nanostructures that, for example, are widely utilized in photonic devices because of their peculiar chemical and physical properties [1,2,3,4,5]. Different dimensions from zero to three-dimensional ZnO nanostructures have been synthesized using various precursors. These nanostructures are particularly important for realizing many applications, such as electronic devices, catalysis, and biomedical and sensing usage, especially, visible ultraviolet optical devices [6,7,8,9,10,11].

Especially, one-dimensional (1D) ZnO nanostructures can be widely used in photonic emitters and photodetectors because of their easy refractive index control, transparency in the visible light range, high photoreactivity, and light waveguide properties [12,13,14]. According to effective medium approximation (EMA), the effective refractive index (*n*_eff_) of ZnO (ZnO film: *n* = 2.1 at visible wavelengths) decreases when it is converted into nanostructures [15,16,17,18].

For achieving a higher photon extraction efficiency (PEE), a material with a refractive index lower than that of ITO (2.1 at visible wavelengths) is required to reduce the total internal reflection (TIR) in a conventional photonic emitter (C-emitter) and increase its outward light emission, that is, in air (*n* = 1). Although the ZnO nanostructures can partially mitigate the abrupt change of refractive indices between *p*-type GaN and air, TIR and Fresnel reflection losses occur at the ZnO/air interface [19,20].

Therefore, alternative materials and structures are required for effective photon extraction from a GaN-based photonic emitter to the outside by matching the refractive indices and for realizing exceptional photon emission from surface nanostructures.

Recently, hierarchical and core–shell nanostructures that provide graded refractive index changes have been applied to achieve high PEE in photonic emitters [11,21,22,23,24,25]. However, for the realization of hierarchical nanostructures, a separate seed layer deposition, high cost vacuum systems, and complicate fabrication processes are required [24,26,27]. To solve these problems, rapid manufacturing techniques are required.

In this study, we demonstrate self-aligned hierarchical ZnO nanorod (ZNR)/NiO nanosheet (NNS) arrays to realize the high PEE of a GaN-based C-emitter. These hierarchical nanostructures are synthesized through a two-step hydrothermal process at low temperatures. The optical output power of the as-obtained C-emitter is approximately 17% and it is two times higher than that of the C-emitter with ZNRs and C-emitter without nanostructures. This increase can be ascribed to a graded change in the refractive index between the GaN layer and the device exterior, as well as a decrease in the TIR and Fresnel reflection of the photonic emitter.

## 2. Materials and Methods

### 2.1. Device Fabrication

Epilayers were grown on a sapphire substrate by metal–organic chemical vapor deposition. The photonic emitter (chip size: 350 × 350 µm^2^) with a 430 nm wavelength consisted of a 0.12 µm-thick *p*-type GaN:Mg (*n* = 3 × 10^17^ cm^−3^) layer, a 0.08 µm active layer, a 2.5 µm-thick undoped GaN layer, and a 4.0 µm-thick *n*-type GaN:Si (*n* = 5 × 10^18^ cm^−3^) layer on the sapphire substrate. All emitter samples were ultrasonically degreased with acetone, methanol, deionized (DI) water, and a mixture of sulfuric acid and hydrogen peroxide (3:1) for 5 min in each step to remove organic and inorganic contaminants. Then, to fabricate the *n*-electrode, the epilayers were partially etched until the *n*-type GaN layer was exposed. The 200 nm thick ITO layer was deposited using an electron-beam evaporator on the remaining parts of the *p*-type GaN layer and annealed at 600 °C in O_2_ atmosphere for 1 min using the rapid thermal annealing. The Ti/Al (50/200 nm) layers were deposited as an *n*-electrode. Finally, the Cr/Al (30/200 nm) layers were deposited on the *p*- and *n*-electrodes and annealed at 300 °C for 1 min.

### 2.2. ZNRs Synthesis

A ZnO seed layer was formed on the selectively deposited ITO by a simple dipping process as follows. First, 105 mM zinc acetate (Zn(C_2_H_3_O_2_)_2_) dissolved in DI water was synthesized at 90 °C for 1 h. Then, ZNRs were grown using 37.5 mM zinc nitrate hexahydrate (Zn(NO_3_)_2_*6H_2_O) and 75 mM hexamethylenetetramine (C_6_H_12_N_4_) dissolved in 300 mL of DI water at 90 °C for 6 h.

### 2.3. Hierarchical ZNR/NNS Arrays Synthesis

Nickel nitrate hexahydrate (Ni(NO_3_)_2_*6H_2_O) (10.4 mg) was dissolved in DI water (50 mL) and stirred for 30 min. Then, this solution was used to synthesis NNSs on the ZNRs at 90 °C for 1 h.

### 2.4. Characterization

The structural shapes of the self-aligned ZNRs and hierarchical ZNR/NNS arrays were observed using a field emission-scanning electron microscope (FESEM, Hitachi S4300, Tokyo, Japan), and the hierarchical nanostructures were analyzed using an energy-dispersive spectroscopy (EDS) mounted on the FESEM. The electrical and optical properties were measured using a parameter analyzer (Keithley 2400, Tektronix, Beaverton, OR, USA), an optical power meter (Newport 1830C, Irvine, CA, USA), and an optical microscope (Velcam CVC5220, Chun Shin Electronics Inc., Taipei, Taiwan). Finite-difference time-domain (FDTD) simulation was conducted to compare the light output of the fabricated photonic emitter with that of a C-emitter.

## 3. Results and Discussion

**Figure 1:**
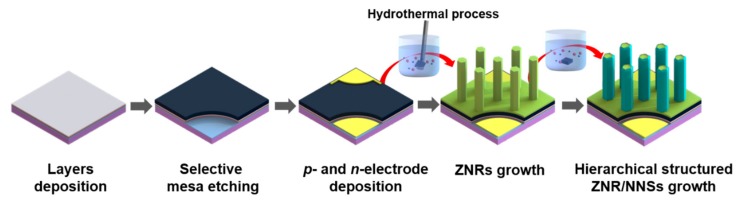
Fabrication process of C-emitter with hierarchical ZnO nanorod (ZNR)/NiO nanosheet (NNS) arrays.

The schematic diagram of the ZNR/NNS arrays synthesis on the C-emitter by following the proposed experimental procedures is shown in Figure 1. 

**Figure 2:**
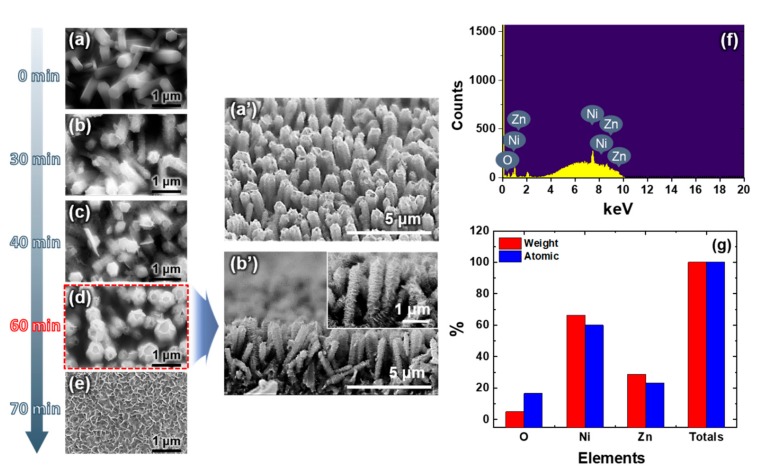
Field emission-scanning electron microscope (FESEM) images of (**a**) ZNRs and (**b**–**e**) hierarchical ZNR/NNS arrays grown for different times; (**a’**) tilt and (**b’**) cross-sectional FESEM images of (**d**). (**f**) an energy-dispersive spectroscopy (EDS) and (**g**) atomic composition of the hierarchical ZNR/NNS arrays.

The FESEM image in Figure 2a shows the ZNRs with an average diameter and length of 300 nm and 3.5 μm, respectively. To understand the growth mechanism of the ZNR/NNS arrays, the study of the morphology evolution of hierarchical ZNR/NNS arrays during the reaction time has been carried out, as shown in Figure 2b–e.

Since the system tends to minimize the overall surface energy, the ZNRs grew preferentially along the [0001] direction [28,29]. Then, Ni-based nanoparticles (NPs) nucleated on the surface of the ZNRs to form active sites, which minimized the interfacial energy barrier to promote the subsequent growth of Ni-based NPs. The merge of these NPs reduced the overall energy by decreasing the surface energy, which was beneficial for adjacent Ni-based NPs to spontaneously self-organize together. The self-organized NPs shared a common crystallographic orientation and formed a planar interface, as shown in Figure 2b. When the reaction proceeded for 30 min, these NPs self-assembled to form large nanosheets (NSs) and finally generated the ZNR/NNS arrays. The above hypothesis is supported by examining the morphologies of NiO NSs at different growth stages by controlling the reaction time, as shown in Figure 2c,d. After exceeding a growth time of 60 min, the NSs transformed into nanowalls (Figure 2e). Therefore, we selected 60 min as the optimum growth time for obtaining stable ZNR/NNS arrays. Figure 2a’,b’ show the tilted and cross-sectional FESEM images of the well-aligned NNS arrays grown for 60 min. The results of an EDS analysis along with the atomic and weight values of the hierarchical ZNR/NNS arrays are shown in Figure 2f. The atomic contents of oxygen, nickel, and zinc were 16.6%, 60.09%, and 23.32%, respectively. The lower percentage of oxygen was ascribed to the fact that oxygen is relatively lighter than nickel and zinc [30]. These results confirmed the successful synthesis of the ZnO and NiO nanostructures.

**Figure 3:**
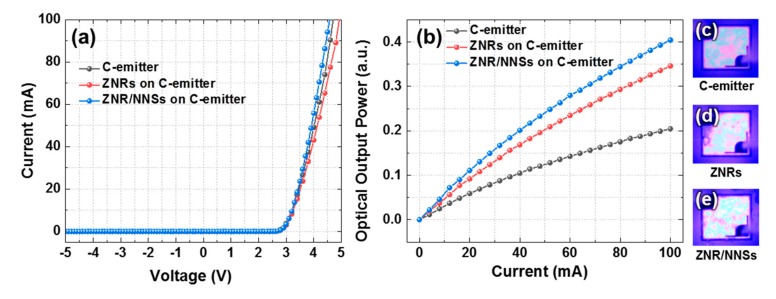
(**a**) Current–voltage curves, (**b**) current–optical output power curves, and (**c**–**e**) emission images (at an injection current of 0.05 mA) of the C-emitter without nanostructures, with ZNRs, and with hierarchical ZNR/NNS arrays.

To evaluate the properties of the fabricated photonic emitters, we measured their current–voltage curves and optical output intensity. Under the injection current of 20 mA, the threshold voltages of the C-emitters without nanostructures, with ZNRs, and with hierarchical ZNR/NNS arrays ranged between 3.00 and 3.03 V, as shown in Figure 3a. This indicates that the ZnO nanostructures did not affect the electrical properties of the emitters because they were grown using a low-temperature growth process. At the injection current of 100 mA, the optical output power of the C-emitter with the hierarchical ZNR/NNS arrays was approximately 17% higher than that of the device with ZNRs and two times higher than that of the photonic emitter without any nanostructures (Figure 3b). The inset emission images in Figure 3c–e show that the hierarchical ZNR/NNS arrays on the C-emitter are brighter than the other emitters at the injection current of 0.05 mA. Compared to other studies on this topic, we realized the optimal refractive index between the ITO layer and air [31,32]. In addition, the higher optical output power can probably be attributed to the graded refractive index and the reduced Fresnel reflection.

**Figure 4:**
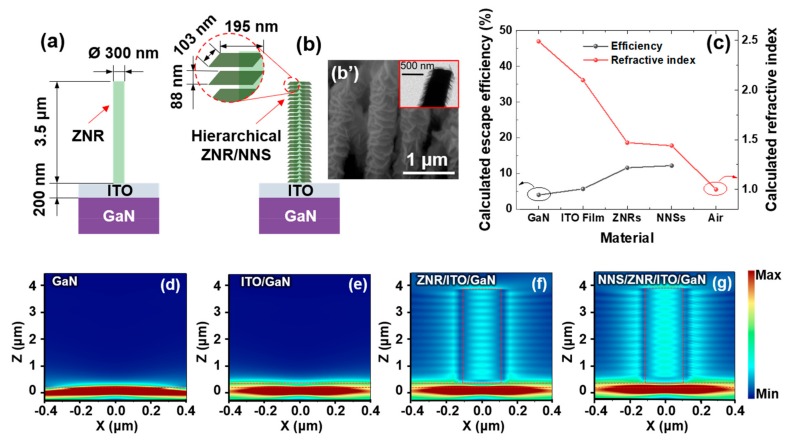
Layouts of the C-emitters with (**a**) ZNR and (**b**) hierarchical ZNR/NNS. (**b’**) FESEM and transmission electron microscope (inset) images of the hierarchical ZNR/NNS arrays. (**c**) Calculated refractive indices and escape efficiencies. (**d**–**g**) Electric field propagation for various C-emitters.

Then, we performed FDTD simulations to compare the three C-emitter types. Figure 4a,b show schematic diagrams of the overall structures of the devices with the ZNR and the hierarchical ZNR/NNS. 

In these simulations, we used the following parameters: ZNR diameter and height of 300 nm and 3.5 μm, respectively, based on the corresponding FESEM image (Figure 2a); NNS width and height of 195 nm and 103 nm, respectively; and consistent interval of 88 nm between adjacent NNSs based on the FESEM and transmission electron microscope results (Figure 4b’). These structures were simulated on the 200 nm thick ITO layer deposited on the 430 nm photonic emitters. The refractive indices of various materials were calculated using EMA [15,16,17]:

(1) $$n_{eff}={\left[n_{ZnO}^{2}V_{ZnO}+n_{Air}^{2}\left(1-V_{ZnO}\right)\right]}^{\frac{1}{2}}$$

where *n*_eff_ is the effective refractive index of the ZNRs; *n*_ZnO_ and n_Air_ are the refractive indices of ZnO and air, respectively; and *V*_ZnO_ is the volume fraction of ZnO in the effective medium. The refractive indices and the volume fraction were determined from the FESEM image, as shown in Figure 2a. The average refractive index of the ZNRs was significantly lower than that of the ZnO film because of the inclusion of air in the effective medium. Therefore, according to EMA, ZNRs have a lower refractive index, even though the refractive index of the ZnO film was similar to that of the ITO film.

Furthermore, the refractive index of NNSs was lower than that of the ZNRs because the refractive index of NiO is lower (1.68) than that of the ZnO film, even though NiO has the lower air volume, as can be inferred from the FESEM image shown in Figure 2d. Therefore, we can confirm that the calculated refractive indices of the GaN, ITO, ZNRs, NNSs are 2.49, 2.1, 1.47, and 1.44, respectively.

The optimal refractive index of the antireflection layer between the ITO film and air can be computed using the formula: [33,34]

(2) $$n_{1}=\sqrt{n_{0}n_{s}}.$$

The formula yielded a value of 1.449, which is very similar to the refractive index of the NNSs for a high antireflection effect.

The escape efficiencies of GaN, ITO, ZNRs, and NNSs (Figure 4c), based on the corresponding calculated refractive indices, can be given as follows [35]:

(3) $$\frac{P_{escape}}{P_{source}}\approx \frac{1}{4}\frac{n_{air}^{2}}{n_{GaN}^{2}}$$

where *n_GaN_* is the refractive index of the GaN layer. *P_escape_* and *P_source_* are the escape and source powers of the photonic emitter, respectively. According to the equation, the photonic emitter without the ITO electrode has an escape efficiency of only 4%. This efficiency can be increased to 12.2% by controlling the refractive index through NNSs growth. However, because this value represents the escape efficiency of photon extraction obtained by considering only the refractive index, it will decrease when considering the efficiency lost through GaN, ITO, and ZNRs. Moreover, the photon efficiency of the device based on various nanostructures is not discussed here, because this calculation considers only layered thin films.

Figure 4d–g illustrate the simulated electric field propagation for the various photonic emitters. As can be inferred from the simulation results shown in Figure 4d,e, the two emitters have similar propagation images. However, the C-emitter shows a higher field extraction toward the outside compared to the emitter without the ITO electrode because of Fresnel reflection. In the case of Figure 4f, although the ZNR on the C-emitter shows a remarkably higher propagation of electric field toward the outside because of the refractive index control and wave guide effect of ZNR, it is limited by the refractive index difference between the ZNR and air and the flat end of the ZNR. This can be explained based on the strong intensity of the electric field inside the ZNR because of interference of the reflected electric field. However, the simulation results of ZNR/NNS on the C-emitter, which are presented in Figure 4g, shows the stronger electric propagation image than that of ZNR on the C-emitter. This difference can be explained by the fact that the NNSs have a smoother graded effective refractive index change and lower TIR loss from the GaN-based photonic emitter to the outside.

## 4. Conclusions

In conclusion, we successfully grew self-aligned hierarchical ZNR/NNS arrays on a C-emitter through a low-temperature two-step hydrothermal process. Compared to the device with only ZNRs, the device with the hierarchical ZNR/NNS arrays exhibited optimal refractive indices (GaN: 2.49, ITO: 2.1, ZNR: 1.47, NNS: 1.44) and antireflection properties as a result of the graded refractive index and waveguide effect. Therefore, at the injection current of 100 mA, its output power was approximately 17% higher than that of the C-emitter with ZNRs and twice as high as that of the device without nanostructures; in addition, there was no degradation of electrical properties. The proposed nanostructures can be used to realize various nanotechnology applications, such as photonic emitters, gas sensors, supercapacitors, electrochromic devices, and solar cells.
